# Consequences of the Loss of the Clicking Mechanism: A Study of Thoracic Functional Morphology in *Plastocerus thoracicus* Fleutiaux, 1918 (Coleoptera: Elateridae)

**DOI:** 10.3390/insects17020212

**Published:** 2026-02-18

**Authors:** Liya Ma, Kexin Sun, Yongying Ruan, Mengna Zhang, Robin Kundrata, Lei Liu, Lu Qiu, Vincent A. D. Hervet, Yang Liu

**Affiliations:** 1 Key Laboratory of Resource Biology and Biotechnology in Western China (Ministry of Education) and College of Life Science, Northwest University, Xi’an 710069, China; maliya2025@outlook.com (L.M.); sunkex2025@outlook.com (K.S.); 15222986176ll@gmail.com (L.L.); 2 Plant Protection Research Center, Shenzhen Polytechnic University, Shenzhen 518055, China; mengnazhang@szpu.edu.cn; 3 Department of Zoology, Faculty of Science, Palacky University, 17. listopadu 50, 771 46 Olomouc, Czech Republic; robin.kundrata@upol.cz; 4 School of Life Sciences (School of Ecological Forestry), Mianyang Teachers’ College, Mianxing West Road, Mianyang 621000, China; 123church@163.com; 5 Agriculture and Agri-Food Canada, Morden Research and Development Centre, Route 100, Unit 100-101, Morden, MB R6M 1Y5, Canada; vincent.hervet@agr.gc.ca; 6 Department of Entomology, University of Manitoba, 12 Dafoe Road, Winnipeg, MB R3T 2N2, Canada

**Keywords:** click beetles, *Plastocerus*, 3D reconstructions, musculature, functions

## Abstract

Elateridae (Coleoptera: Elateroidea) are renowned for their clicking mechanism. However, several lineages exhibit body softening that compromises this mechanism, particularly within Plastocerini, Drilini, and Omalisinae. It remains unclear how this body softening is anatomically achieved and which specific structures are degraded in relation to the loss of clicking function. To elucidate the internal morphological adaptations and distinguish them from hard-bodied clicking elateroids, we employed micro-CT to scan *Plastocerus thoracicus* and reconstruct its thoracic morphology in 3D and quantified key muscle ratios (e.g., M2/M60, M4/M60). Based on our study of *P. thoracicus,* a detailed comparison was made with previously reported data on *Campsosternus auratus* (Elateridae) and *Cerophytum lii* (Cerophytidae). Three-dimensional reconstructions revealed significant structural divergences in *P. thoracicus*: (1) the clicking-related muscles M4 are markedly weaker than those in *Ca. auratus* and *Ce. Lii*. (2) the prosternal process (PP) is extremely narrow. The posterior part of the pronotum exhibits underdeveloped regions, including the posterodorsal evagination (PdE) and the posteromedial process (PmPr). (3) the mesonotum (i.e., the “biological spring” identified in previous studies) is greatly flattened and weakened. (4) the flight muscles (M60, M64) and walking muscles (M74, M75) exhibited significantly bigger volume than *Ca. auratus* and *Ce. lii*. These findings provide critical data for understanding the morphological evolution of Elateridae and offer insights into the functional adaptations of the clicking mechanism through comparative anatomy.

## Simple Summary

Chinese Plastocerini (Elateridae: Dendrometrinae) are represented by a single and rare species *Plastocerus thoracicus* Fleutiaux, 1918, which exhibits cuticle softening and the loss of the functional clicking mechanism. Here, we employed micro-computed tomography (micro-CT) and three-dimensional (3D) reconstruction to elucidate the thoracic morphology of this species. While body softening has been documented in several Elateridae groups, the associated internal structural modifications remained unexplored. Specifically, this study aimed to identify the anatomical changes in the thoracic structures associated with body softening and the loss of the clicking mechanism. By conducting comparative morphological analyses between *P. thoracicus* and two functionally contrasting species, *Campsosternus auratus* (Drury) and *Cerophytum lii* Qiu & Ruan, we provide insights into this question.

## 1. Introduction

Elateridae, a diverse group within Coleoptera, comprise approximately 14,600 described species worldwide [1], with 1516 species recorded in China [2]. Elateridae are well known for their ability to jump with the typical clicking sound using the pro-mesothoracic clicking mechanism [3,4], which gave them their common name, click beetles. Although Elateridae traditionally included almost exclusively hard-bodied species which possessed clicking mechanism [5], after recent dramatic changes in Elateroidea classification, this family also includes lineages with a soft body and non-functional or even absent clicking mechanism [1,6,7].

*Plastocerus* Schaum, 1852, looks in many aspects like the majority of hard-bodied click beetles. However, it exhibits some skeletal softening together with weakened clicking-related structures, visible protrochantins, and more freely articulated abdominal ventrites, with some or all abdominal segments free, depending on the sex [6,8,9]. Therefore, *Plastocerus* is morphologically intermediate between typical, hard-bodied click beetles and soft-bodied lineages with an absent clicking mechanism and free, extremely movable abdomen (e.g., Omalisinae, Agrypninae: Drilini) [1]. Taking into account the modified morphology of *Plastocerus*, it is not surprising that its taxonomic placement has long been contentious [9,10]. Initially, Schaum [11] assigned it to the family Cebrionidae. Later, it was classified either in Elateridae [12,13] or its own family Plastoceridae within Cantharoidea [8], which were later merged under Elateroidea. Based on the results of molecular phylogenetic analyses, Bocak et al. [6] provided critical evidence that *Plastocerus* (Plastocerinae) is in Elateridae and found it being a sister group to Oxynopterini (or Oxynopterinae by some). Douglas et al. [7] conducted a comprehensive phylogenomic study that reclassified Plastocerinae as the tribe Plastocerini under the subfamily Dendrometrinae. Currently, *Plastocerus* contains two species, i.e., *P. angulosus* (Germar, 1844), from Europe and West Asia, and *P. thoracicus* Fleutiaux, 1918, from East and Southeast Asia [9,10,14]. The latter species is rare in China [15].

The modified morphology of *Plastocerus thoracicus* with reduced pro-mesothoracic structures contrasts sharply with two previously studied elateroid species, *Campsosternus auratus* [4] and *Cerophytum lii* [16], which exhibit fully functional clicking mechanisms. This study employed micro-computed tomography (micro-CT) and three-dimensional (3D) reconstruction to investigate the thoracic structural changes in *P. thoracicus*. Specifically, we aimed to elucidate the anatomical changes associated with body softening and to identify the muscles and sclerites linked to the loss of the functional clicking mechanism.

## 2. Materials and Methods

### 2.1. Micro-CT Scanning and 3D Reconstructions

A needle-mounted dry specimen of *Plastocerus thoracicus* was subjected to micro-computed tomography (micro-CT) scanning. The dried specimen was placed in a cylindrical specimen holder and stabilized with transparent plastic film to minimize movement artifacts during image acquisition. The fixed specimen was scanned using a Scanco Medical μCT100 scanner (Scanco Medical Inc., Wangen Bruttisellen, Switzerland) under the following parameters: X-ray voltage 45 kV, current 200 μA, and power 9 W; voxel size 1.2 μm; field of view (FOV) 9.828 mm; absorption contrast with 360 steps; and image matrix 8190 × 8190 pixels. A total of 6936 cross-sectional images were reconstructed from the acquisition. The μCT image slices were imported into Avizo 2020 for preliminary processing. Extraneous debris surrounding the specimen were digitally cropped, and the HDR-formatted data were converted into pvl.nc format using Drishti Impart (Drishti 4.0) [17]. The 3D model was reconstructed in Drishti 4.0, wherein sclerites and muscles were segmented and color-coded within the Paint module, followed by final surface rendering in the same software. The final compositional layout for figures was assembled using Adobe Photoshop 2019.

The specimen used for 3D reconstruction in this paper is currently preserved at the Institute of Zoology, Chinese Academy of Sciences (IZCAS, Beijing, China). The classification of Elateroidea and Elateridae follows Kundrata [1].

Sample information includes the following: *Plastocerus thoracicus* Fleutiaux, 1918 [18]; collected in Shangcheng County, Xinyang City, Henan Province, Jinggangtai, on 26 June 2006, Ye Liu leg. (Institute of Zoology, Chinese Academy of Sciences; IZCAS, Beijing). Measurements included the following: body length 7.17 mm; body width 2.24 mm; pronotum length 1.40 mm; and pronotum width 1.74 mm.

Muscle volume is obtained in Drishti 4.0 using the “Volume function” in “Command Help” or in Avizo 2020 using “Surface View” and the “Surface Area Volume” function.

### 2.2. Terms and Abbreviations

Morphological terms are based on Ruan et al. [4], Lawrence et al. [19], and Douglas [20]; and muscle names follow those of Larsén [21]:

**1AT……8AT** = abdominal tergites 1–8; **1Pm/2Pm/3Pm** = first/second/third phragma of the thorax; **3ASt……7ASt** = abdominal sternites 3–7, equivalent to abdominal ventrites 1–5; **3Pm-ML**/**3Pm-LP** = median lobe/lateral process of the third phragma; **Abd** = abdomen; **Act** = acetabulum; **Alc** = alacrista of the metanotum [22]; **AmE** = anteromedium emargination of the mesonotum; **APm** = abdominal ventral phragma [23,24]; **AR** = anterolateral region of the mesonotum, with a highly smooth surface; **PaBr** = prealar bridge of the mesonotum, also known as the prealar arm in [22]; **AWP2/AWP3** = anterior notal wing process of the mesonotum/metanotum; **Ax1**/**Ax2**/**Ax3** = (first, second, and third) axillary sclerite; **AxC** = axillary cord; **Ba** = basalar sclerite; **BaD** = basalar disc; **BEL** = basal lobe of the elytron [21], also as the ‘elytral root’ in [25,26]; **Cl** = anterior collar of the pronotum (i.e., inflected anterior margin of the pronotum); **Crpl** = cryptopleuron, equivalent to the endopleuron; **Cu** = elastic cuticle; **Cv1/Cv2** = cervical sclerite 1/2; **Cx1**/**Cx2**/**Cx3** = pro-/meso-/metacoxa; **CxP** = metacoxal plate; **CxR** = procoxal rest of the mesoventrite [27]; **Dc** = metathoracic discrimen [27]; **Ely** = elytron; **Em2/Em3** = mesepimeron/metepimeron; **Epi** = elytral epipleuron; **Es2/Es3** = mesanepisternum/metanepisternum; **F1** = prothoracic furca; **F2** = mesothoracic furca, equivalent to the mesendosternite; **F3** = metathoracic furca, equivalent to the metendosternite; **FB** = profurcal base or prosternal furcal base, also known as the ‘bumper’ in [3]; **FH** = friction hold [3], a lowered area on the posterodorsal end of the prosternal process, also known as the ‘peghold’ in [3]; **FP** = furcal pit; **H** = head; **Hy** = hypomeron; **I** = insertion of muscle; **IAM** = inflected anterior margin of the mesonotum; **LA** = lateral arm of the furca [28]; **LC** = lateral carina [29]; **LC-i** = internal trace of the lateral carina; **LGr** = lateral groove of the mesonotum; **PRM** = prosternal rest of the mesoventrite [27], i.e., the anteromedian extension of the mesoventrite, also known as the ‘mesosternal lip’ and ‘lip of the mesosternum’ in [3]; **M1/M2……M85** = muscles 1–85 [22]; **MAr** = median-arched area of the mesonotum; **Meb** = membrane; MGr = median groove of the metanotum; **MRMs** = median ridge of the metaventrite; **MsC** = mesoventral cavity [27], also known as the ‘mesosternal cavity‘ [29] and the ‘mesosternal fossa’ [30]; **N I/N II/N III** = pro-/meso-/metanotum; **O** = origin of muscle; **PA** = posterior angle of the pronotum; **PCP** = pleural coxal process; **PdE/PvE** = posterodorsal/posteroventral evagination of the pronotum; **PdEB** = the anteromesal bulge part of the **PdE**, sensu the ‘knob‘ on the underside of the posterior margin of the pronotum in [3]; **PE** = posterior evaginations of the pronotum [21], also known as the ‘pronotal flange’ in [3], consisting of the **PdE** and **PvE**; **PGr** = posterodorsal groove of the pronotum, situated above the posterodorsal evagination; **PlA** = pleural arm of the meso-/metapleuron; **PlR** = pleural ridge of the meso-/metapleuron; **PlS** = pleuro suture; **PlWP2/PlWP3** = pleural wing process of the mesothorax/metathorax; **PmPr** = posteromedial part of the pronotum; **Pn3** = postnotum of the metathorax; **PP** = prosternal process; **Pra** = prealar sclerite of the metathorax, consisting of an externally visible isolated sclerite and the internal mushroom-shaped plate; **Prs3** = metathoracic prescutum; **PSA** = pronotosternal articulation; **PScl2** = posterior scutellum of mesonotum; **PsS** = pronotosternal suture; **Sa** = subalar sclerite; **Scl2/Scl3** = meso-/metascutellum; **SclS2** = mesoscutellar shield; **Sct2/Sct3** = mesoscutum/metascutum; **SpS** = sternopleural suture; **St I** = prosternum; **Stk** = stalk of the metathoracic furca [28], equivalent to the basal part of the metaendosternite; **Tn** = trochantin; **Tr** = trochanter; **VF** = ventral flange of the metafurca; **Vt II/Vt III** = mesoventrite/metaventrite [27], known as the mesosternum/metasternum in earlier works; and **YP** = yoke plate [22].

## 3. Results

### 3.1. Functional Morphology of the Thorax of Plastocerus thoracicus

#### 3.1.1. General Morphology (Figure 1 and Figure 2)

In dorsal view, the body is elongated, nearly parallel-sided, and slightly tapering near the anterior and posterior ends; in lateral view, the body is flattened dorsoventrally. The body surface bears soft, fine setae and is covered with coarse punctures, lacking metallic luster.

The thoracic exoskeleton is less sclerotized compared to click beetles with a full clicking ability (e.g., *Campsosternus auratus*). The thorax exhibits an extremely narrow prosternal process, a weakly developed mesoventral cavity, and a well-developed pronotal posterior angles. The mesonotum is much more flattened than other click beetles and is not specialized into a strongly curved shape. The posterior part of the prothorax does not fit well with the anterior part of the mesothorax. The clicking mechanism is absent, and the promesothoracic interlocking mechanism is weak.

**Figure 1:**
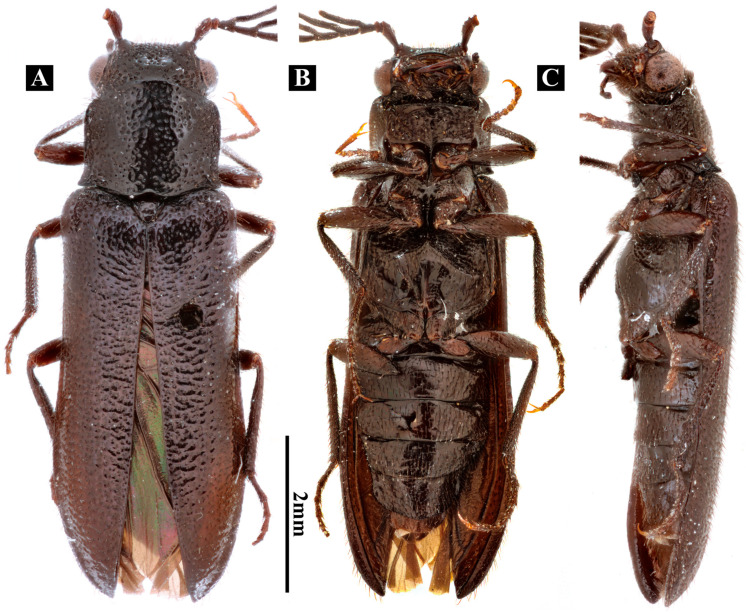
Habitus of *Plastocerus thoracicus*. (**A**): Dorsal view. (**B**): Ventral view. (**C**): Lateral view.

**Figure 2:**
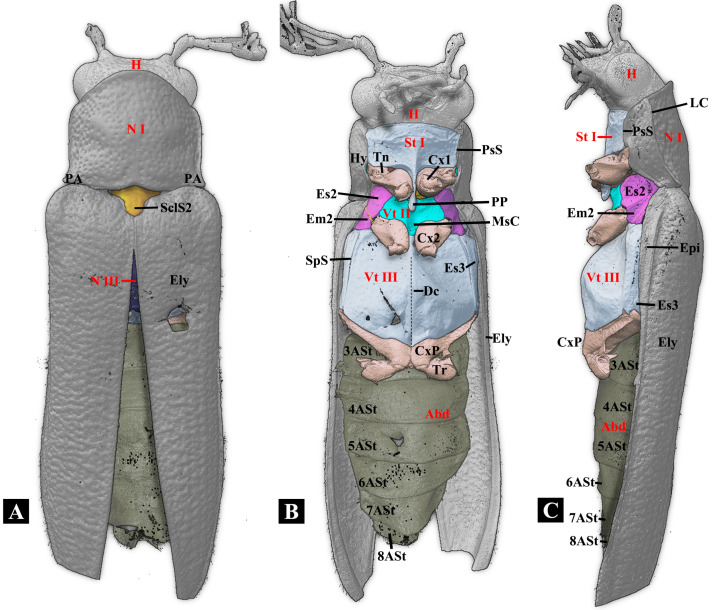
Three-dimensional reconstructions of the exoskeleton of *Plastocerus thoracicus*. (**A**): Dorsal view. (**B**): Ventral view. (**C**): Lateral view. For technical abbreviations, see Section 2.

#### 3.1.2. Head (Figure 1 and Figure 2)

The head is flattened dorsoventrally and prognathous. It is approximately the same width as the anterior part of the body, bears chewing mouthparts, and has oval-shaped compound eyes with pectinate antennae. The posterior part (postocciput) is connected to the prothorax via cervical membranes. The following muscles are connected to the posterior part of the head: M1, M2b, M5, M6, M7, M8, and M10.

**Antennae:** 11 segments, pectinate, with rami beginning at the 3rd antennomere. **Subantennal groove:** Absent. **Supra-orbital groove:** Absent. **Antennal insertion cavity (observed from above):** Concealed by a ridge. **Mandibular apex:** Unidentate. **Labrum:** Exposed, weakly developed, band-like, visible in fronto-ventral view but concealed by the frontoclypeal region in frontal view. **Supra-antennal carina (SC):** Circular, well-developed surrounding antennal fossa; no split adjacent to the eyes.

#### 3.1.3. Prothorax

**Figure 3:**
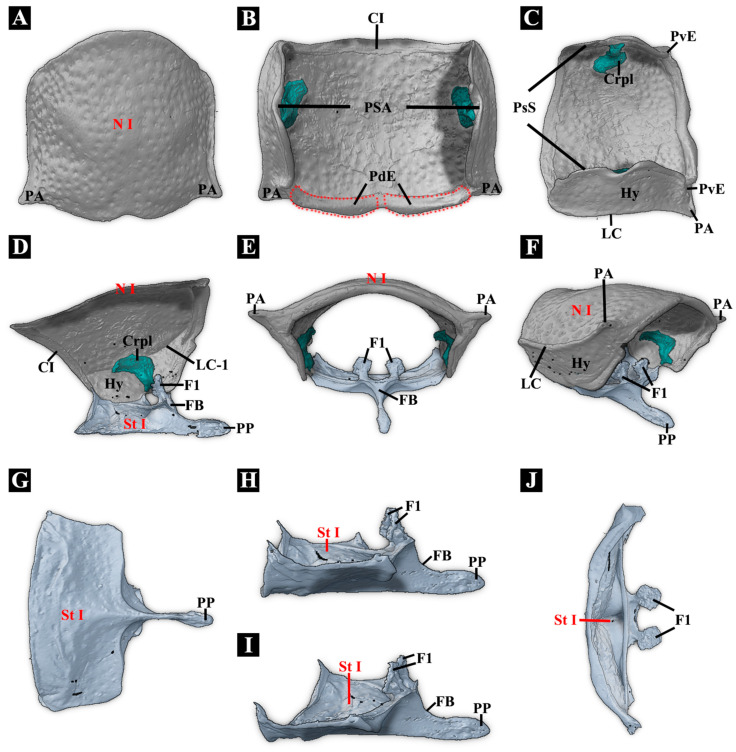
Three-dimensional reconstructions of the prothoracic exoskeleton of *Plastocerus thoracicus*. (**A**–**C**): Dorsal, ventral, and lateral views of the pronotum. PdE is marked with a red dashed line. (**D**): Mesal view of the prothorax cut in the sagittal plane. (**E**–**F**): Caudal and posterolateral view of the prothorax. (**G**–**J**): Dorsal, lateral, and frontal views of the prosternum. For abbreviations, see Section 2.

**(1)**  **Pronotum** (Figure 3A–F)

**Pronotum (N I):** Nearly rectangular in lateral view, dorsum slightly arched. **Collar:** Present and well-sclerotized. **Lateral pronotal carinae (LC-e):** Complete. **Sublateral longitudinal carinae and groove:** Absent. **Pronotum with posterior sublateral incisions** (see Ruan [31] for definition): Absent. **Posteromedian part of pronotum (PmPr):** In dorsal view, PmPr slightly expands posteriorly, with a concave, smooth ventral surface; in ventral view, the ventral surface is weakly developed. **Posterior edge of pronotum with concavity mesad of hind angles in dorsal view:** Absent. **Posterodorsal evagination of pronotum (PdE):** Present, slightly raised, not prominent. **Posteroventral evagination of pronotum (PvE):** Present. PvE is the evaginated part of the posterior margin of the hypomeron. It is weakly developed. **Posterodorsal groove (PGr):** Absent. **Posterior angle of the prothorax (PA):** Weakly developed, not produced posterolaterally. In typical Elateridae, PA is usually wedge-shaped, well-sclerotized, and produced posterolaterally.

**(2)**  **Cryptopleuron** (Figure 3D)

**Cryptopleuron (Crpl):** Well-developed. In some Elateridae, the propleuron is invisible externally and reduced to the cryptopleuron, which is mushroom-shaped, with M16 and M20 attached. The ventral part of Crpl is partly merged with the trochantin. **Propleurotrochantin (Tn):** Well-developed.

**(3)**  **Prosternum** (Figure 3C–J)

**Prosternal process (PP):** In ventral view, the PP is extremely narrow and unable to sustain great stress and thus cannot be used for clicking. It is extremely thin between the procoxae, with the procoxae almost in contact with each other and slightly dilated near the apex. In the lateral view, it is straight and not curved dorsally. **Friction hold (FH):** Absent. **Profurca (F1):** F1 is fan-shaped, with M5, M6, M30, and M11 attached. The furcal pit is visible in ventral view when the procoxa is removed. **Pronotosternal suture (PsS)**: Straight. The prosternum and hypomeron are entirely fused, and the anterior part of the PsS is immobile. The PsS is not prominent. **Prosternum with paired carinae:** Absent. **Prosternal chin piece:** Short and weakly developed. **Prosternal head rest** (infolded anterior edge of the prosternum)**:** Moderately broad, horizontal to slightly oblique.

#### 3.1.4. Mesothorax

**Figure 4:**
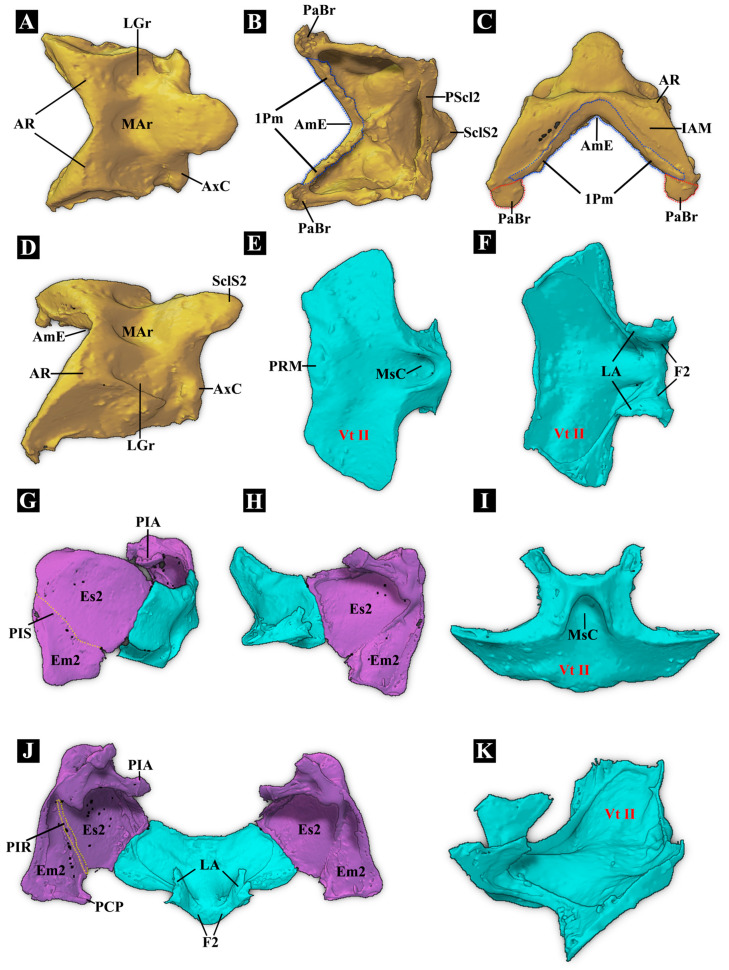
Three-dimensional reconstructions of the mesothoracic exoskeleton of *Plastocerus thoracicus*. (**A**–**D**): Dorsal, ventral, frontal, and anterolateral views of the mesonotum: PaBr is marked with a red dashed line, and 1 Pm is marked with a blue dashed line. (**E**,**F**,**I**,**K**): Ventral, dorsal, frontal, and lateral views of the mesoventrite. (**G**): External view of the left mesopleuron: PIS is marked with a yellow dashed line. (**H**): Internal view of the left mesopleuron. (**J**): Dorsal view of the mesopleurae: PIR is marked with a yellow dashed line. For technical abbreviations, see Section 2.

**(1)**  **Mesonotum** (Figure 4A–D)

**Mesonotum (N II):** Not specialized into a saddle-shaped structure, weakly sclerotized. **Mesoscutellar catch:** Weakly developed. **Anteromedian emargination of mesonotum (AmE):** Present and V-shaped. **Anterolateral region of mesonotum (AR):** Weakly developed. It is located on both lateral sides of the anterior part of the mesonotum. Compared to that in *Campsosternus auratus*, it exhibits a significantly reduced surface area and a loss of its characteristic semicircular morphology. This structural impairment prevents the formation of a functional “thoracic hinge” with the posterior dorsal emargination (PdE). **Prealar bridge of mesonotum (PaBr):** Triangular and well-sclerotized. M4x is attached to PaBr, which connects to the anterolateral side of the mesoscutum via elastic cuticle. **First phragma (1Pm):** Present, located at the anteroventral part of the mesonotum. M4 inserts at the middle part of 1Pm, while muscles M8 and M11 attach to the anterolateral part of 1Pm. **Mesoscutellar shield (SclS2):** SclS2 forms a slightly slanted slope, with the anterior part lowered and gradually merging with the posterior part of MAr; the posterior part is slightly elevated. The anterior part of SclS2 is much lower in this species than in other elaterids; whereas in other elaterids, it is usually vertical or inflected. **Median arched area of mesonotum (MAr):** Weakly developed, slightly concave. **Mesoscutellum (Scl2):** Weakly developed. **Mesoscutum (Sct2):** Weakly developed.

**Scutellum with anterior edge:** Merges and forms a slope with MAr, featuring a continuous smooth surface. **Posterior edge of scutellum:** Convex. **Yoke plate (YP):** Obsolete. The inability of YP to make contact with the metathoracic prescutum (Prs3) on the metanotum could be one of the reasons for the failure to perform the clicking mechanism.

**(2)**  **Mesopleuron** (Figure 4G,H,J)

**Mesanepisternum (Es2)** and **Mesepimeron (Em2):** Well-developed. **Mesopleural suture (PlS) and mesopleural ridge (PlR):** Well-developed.

**(3)**  **Mesoventrite (Vt II)** (Figure 4E,F,I,K)

**Prosternal rest of mesoventrite (PRM):** Weakly developed. Incised at its anterior margin and V-shaped. **Mesoventral cavity (MsC):** Extremely shallow and unable to serve as a component for clicking. **Meso-metaventral junction**: Obscure. **Distance between mesocoxal cavities:** Less than half the shortest diameter of the coxal cavity. **Mesothoracic furca (F2):** Well-developed.

#### 3.1.5. Metathorax

**Figure 5:**
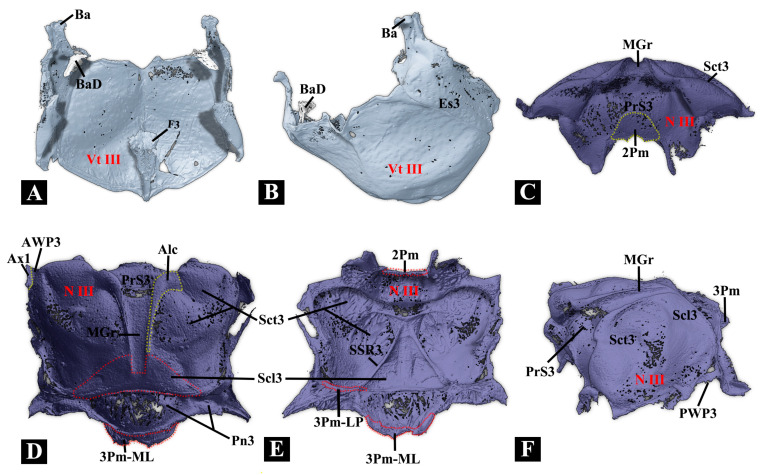
Three-dimensional reconstructions of the metathoracic exoskeleton of *Plastocerus thoracicus*. (**A**–**B**): Dorsal and lateral views of the metaventrite. (**C**–**F**): Frontal, dorsal, ventral, and lateral views of the metanotum. For technical abbreviations, see Section 2.

**(1)**  **Metanotum (N III)** (Figure 5A,B)

The metanotum is very weakly sclerotized. Its median border is loosely connected to the mesonotum by a flexible membrane, while the lateral sides feature the **metathoracic prescutum (Prs3)** closely attached to the mesonotal yoke plate via an inflexible elastic cuticle. Additionally, the lateral and posterior borders of the metanotum are connected to the metapleuron and abdominal tergite, respectively, through loose, flexible membranes. Notably, the anterolateral area of the metanotum bears the axillary sclerite and hind wing.

**(2)**  **Metapleuron (Pl III)** (Figure 5A,B)

The metapleuron consists of the **metanepisternum (Es3)** and the **metepimeron (Em3)**. The Es3 is well-sclerotized and somewhat wedge-shaped, with its anterodorsal part strongly produced, bearing the knob-like metathoracic **pleural wing process (PlWP3)** and the **basalar sclerite (Ba).** In the internal view, the Ba is well-developed and produced posteroventrally, forming a basalar disc at its posterior end. The Em3 is weakly sclerotized and elongated; its dorsal part slightly turns towards the metanotum, and a flexible membrane connects the two structures.

**(3)**  **Metaventrite (Vt III)** (Figure 5C–F)

The metaventrite is strongly sclerotized, with a quadrate general shape. In ventral view, the anterior margin is somewhat fused with the mesoventrite. The median ridge is strongly developed and well-sclerotized. **Metaventral discrimen (Dc):** Long, extending anteriorly beyond the middle of the ventrite. **Metaventral postcoxal lines:** Absent. **Metaventrite:** Without grooves for reception of tarsi. **Exposed portion of metanepisternum:** More than five times as long as wide. **Internal trochantinal disc of metathorax:** Well-developed, umbrella-shaped, and connected to M73. **Metathoracic furca (F3):** LA absent, forming an overall triangular prism shape with an enlarged end.

#### 3.1.6. Legs

**Protrochanter and Mesotrochanter:** More than twice as long as wide. **Mesocoxae:** Not open to the Es2 and Em2, enclosed by the meso- and metasternum. **Metacoxae:** Not separated. **Metacoxa:** Strongly transverse. **Metacoxal plate:** Weakly developed. **Protibia:** Protibia, mesotibia, and metatibia with two apical spurs each. **Outer edge of mesotibia:** Without a sharp carina. **Pretarsal claws:** Simple, neither serrate nor pectinate. **Membranous ventral lobes on mesotarsus:** Absent.

#### 3.1.7. Thoracic Musculature

We follow the muscle nomenclatures (M1 to M85) by Larsén [21]; the muscle nomenclature used by Friedrich and Beutel [32] is put in parentheses. In the following, O and I are abbreviations for origin and insertion of muscle, respectively.

**(1)**  **Prothoracic musculature**

**M1 (Idlm2):** *M. pronoti primus.* O: anteromedian part of pronotum, I: dorsolateral part of postoccipital ridge. Y-shaped, flat, slender.

**Figure 6:**
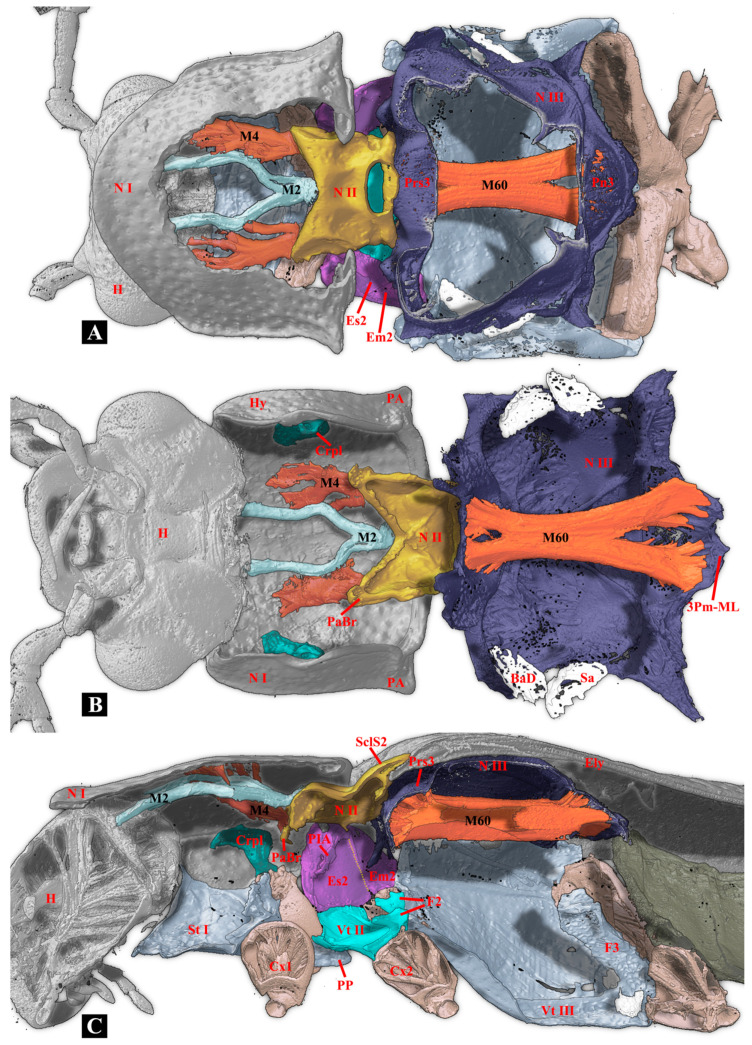
Three-dimensional reconstructions of the thoracic exoskeleton and musculature of *Plastocerus thoracicus*. The specimen is in a resting position; for technical information and abbreviations, see Section 2. (**A**): Dorsal view. (**B**): Ventral view. (**C**): Lateral view.

**M2a (Idlm1):** *M. pronoti secundus*. O: median dorsal apex of first phragma (1Pm), I: dorsolateral part of postoccipital ridge. (Figure 6A–C).

**M2b (Idlm1):** *M. pronoti secundus.* O: median dorsal apex of first phragma (1Pm), I: collar of pronotum (inflected anterior margin of pronotum). The M2 (M2a+M2b) are long, robust, and Y-shaped. (Figure 6A–C).

**M4 (Idlm5):** *M. pronoti quartus*. O: major area of pronotum, I: anterolateral part of the first phragma (1Pm). Weakly developed. (Figure 6A–C).

**M4x (Idlm5):** O: posterior part of pronotum (N I), I: prealar bridge of mesonotum (PaBr). M4x was not mentioned for Elateridae by Larsén [21]. Fan-shaped.

**M5 (Ivlm3):** *M. prosterni primus*. O: profurcal arm (F1), I: posterior tentorial arm of the head. Well-developed, longitudinal.

**M6 (Ivlm1):** *M. prosterni secundus.* O: profurcal arm (F1), I: ventral part of the cervical membrane and the anterior cervical sclerite (Cv1). Well-developed, longitudinal.

**M7 (Idvm6):** *M. dorsoventralis primus*. O: anterolateral region of pronotum (N I), I: ventrolateral part of cervical membrane. Flat, slender, longitudinal.

**M8 (Idvm8):** *M. dorsoventralis secundus.* O: lateral part of the first phragma (1Pm), I: dorsolateral part of the postoccipital ridge. Slender, longitudinal.

**M10 (Idvm2, 3?):** *M. dorsoventralis quartus.* O: anterolateral part of prosternum (St I), I: dorsal part of postoccipital ridge.

**M11 (Idvm10):** *M. dorsoventralis quintus.* O: profurcal arm (F1), I: lateral part of the first phragma (1Pm). Slender, flat.

**M12 (Itpm3):** *M. noto-pleuralis*. O: posterolateral part of pronotum (N I), I: anterior part of the cryptopleuron (Crpl). A broad but mostly short muscle.

**M15 (Idvm16, 17):** *M. noto-coxalis*. O: posterolateral part of pronotum (N I), I: process of procoxa (Cx1). Well-developed, conical.

**M16 (Ipcm4):** *M. episterno-coxalis*. O: anterior part of the cryptopleuron (Crpl), I: process and rim of procoxa (Cx1). Well-developed, fan-shaped.

**M19 (Iscm2):** *M. furca-coxalis*. O: profurcal arm (F1), I: process of the procoxa (Cx1). Short and small.

**M20 (Ipcm8):** *M. pleura-trochanteralis*. O: posterior part of the cryptopleuron (Crpl), I: trochanter of the proleg. Well-developed, fan-shaped.

**(2)**  **Mesothoracic musculature**

**M28 (IIdlm1):** *M. mesonoti primus*. O: posterior part of the first phragma (1Pm), I: dorsal part of the second phragma (2Pm). Well-developed, slightly fan-shaped.

**M29 (IIdlm2):** *M. mesonoti secundus.* O: posterior part of the first phragma (1Pm), I: anterolateral part of metathoracic prescutum (Prs3). Well-developed, fan-shaped.

**M30 (Ivlm7):** *M. mesosterni primus.* O: proforcal arm (F1), I: mesofurcal arm (F2). Well-developed, long, and cylindrical.

**M32x (IIdvm8):** *M. dorso-ventralis.* O: mesothoracic axillary sclerites (?), I: lateral inflected area of mesoventrite (Vt II). Slender and cylindrical.

**M33 (IItpm2):** *M. noto-pleuralis*. O: first phragma (1Pm), I: pleural arm of mesopleuron (PlA). Well-developed, short, and cylindrical.

**M36 (IItpm9):** *M. pleura-alaris*. O: pleural arm of mesopleuron (PlA), I: third axillary sclerite of mesothorax (Ax3). Extremely weakly developed, cylindrical. (Owing to space constraints, the M36 muscle is not illustrated on the plate).

**M37 (IIspm2):** *M. furca-pleuralis*. O: mesofurcal arm (F2), I: lower part of the pleural ridge of mesopleuron (PlR). Weakly developed, small.

**M40 (IIdvm4, 5):** *M. noto-coxalis.* O: posterolateral part of mesonotum (N II), I: posterior rim of mesocoxa (Cx2). Well-developed.

**M41 (IIpcm4):** *M. episterno-coxalis.* O: mesanepisternum (Es2), I: anterolateral rim of mesocoxa (Cx2). Well-developed, fan-shaped.

**M45 (IIscm4):** *M. furca-coxalis lateralis.* O: mesofurcal arm (F2), I: posterolateral rim of mesocoxa. Weakly developed, small.

**M46 (IIscm2)**: *M. mesofurca-coxalis posterior.* O: mesofurcal arm (F2), I: posterior rim of mesocoxa (Cx2). Weakly developed, small.

**M48 (IIpcm6):** *M. episterno-trochanteralis.* O: mesanepisternum (Es2), I: trochanteral tendon. Well-developed, fan-shaped.

**(3)**  **Metathoracic musculature**

**M60 (IIIdlm1):** *M. metanoti primus*. O: second phragma (2Pm) and middle part of metathoracic prescutum (Prs3), I: median lobe of third phragma (3Pm-ML) and postnotum (Pn3). Strongly developed and cylindrical (Figure 6A–C).

**M61 (IIIdlm2):** *M. metanoti secundus*. O: middle part of metascutum (Sct3), I: lateral process of third phragma (3Pm-LP). Well-developed, oblique, and cylindrical.

**M64 (IIIdvm1):** *M. dorsoventralis primus.* O: median part and median ridge of the metaventrite, I: metathoracic prescutum (Prs3). Strongly developed, oblique, and cylindrical.

**M66 (IIIdvm8):** *M. dorsoventralis tertius.* O: lateral arm of metafurca (LA), I: lateral process of third phragma (3Pm-LP). Slender, small.

**M67 (IIItpm2):** *M. pleura-praealaris.* O: prealar sclerite (Pra), I: pleural ridge of metapleuron (PlR). Small, conical, and fan-shaped.

**M69 (IIItpm3):** *M. noto-basalaris*. O: lateral part of the metathoracic prescutum (Prs3), I: basalar disc (BaD). Well-developed, short, and cylindrical.

**M71 (IIItpm7, 9):** *M. pleura-alaris.* O: metanepisternum (Es3), I: a small sclerite in the membrane under the third axillary sclerite. Triangular, short, small.

**M73 (IIIspm1):** *M. sternobasalis*. O: lateral part of metaventrite (Vt III), I: basalar disc (BaD). Well-developed, cylindrical.

**M74 (IIIdvm2):** *M. noto-trochantinalis*. O: anterior part of metascutum (Sct3), I: trochantinal disc. Strongly developed, cylindrical.

**M75 (IIIdvm4):** *M. noto-coxalis anterior.* O: middle part of metascutum (Sct3), I: inner surface of metacoxa (Cx3). Strongly developed, cylindrical.

**M76 (IIIdvm5):** *M. noto-coxalis posterior.* O: lateral margin of metascutum (Sct3), I: inner surface of metacoxa (Cx3). Slender and cylindrical.

**M78 (IIIpcm3):** *M. coxa-basalaris.* O: anterior margin of metacoxa (Cx3), I: basalar disc (BaD). Slender, cylindrical.

**M79 (IIIdvm6):** *M. coxa-subalaris*. O: inner surface of metacoxa (Cx3), I: subalar disc (Sa). Well-developed, cylindrical.

**M81 (IIIscm1):** *M. furca-coxalis anterior.* O: stalk of the metafurca (Stk), I: anteromesal rim of metacoxa (Cx3). Well-developed, short.

**M82 (IIIscm4):** *M. furca-coxalis lateralis.* O: ventral flange of metafurca (VF), I: a process on the anterolateral rim of the metacoxa (Cx3). Well-developed, transverse, long, and conical.

**M83a (IIIscm2):** *M. metafurca-coxalis posterior*. O: dorsal surface of the lateral arm of the metafurca (LA), I: posterior rim of the coxa (Cx3). Well-developed, broad, and flattened.

**M83b (IIIscm3):** *M. metafurca-coxalis posterior.* O: ventral surface of the stalk of the metafurca (Stk), I: mesal part of the posterior rim of the metacoxa (Cx3). Well-developed and conical.

**M85 (IIIscm6):** *M. furca-trochanteralis.* O: lateral arm of the metafurca (LA), I: trochanteral tendon. Well-developed, slender, and cylindrical.

### 3.2. Comparative Morphology: Thoracic Exoskeleton of Campsosternus auratus, Cerophytum lii, and Plastocerus thoracicus

**Figure 7:**
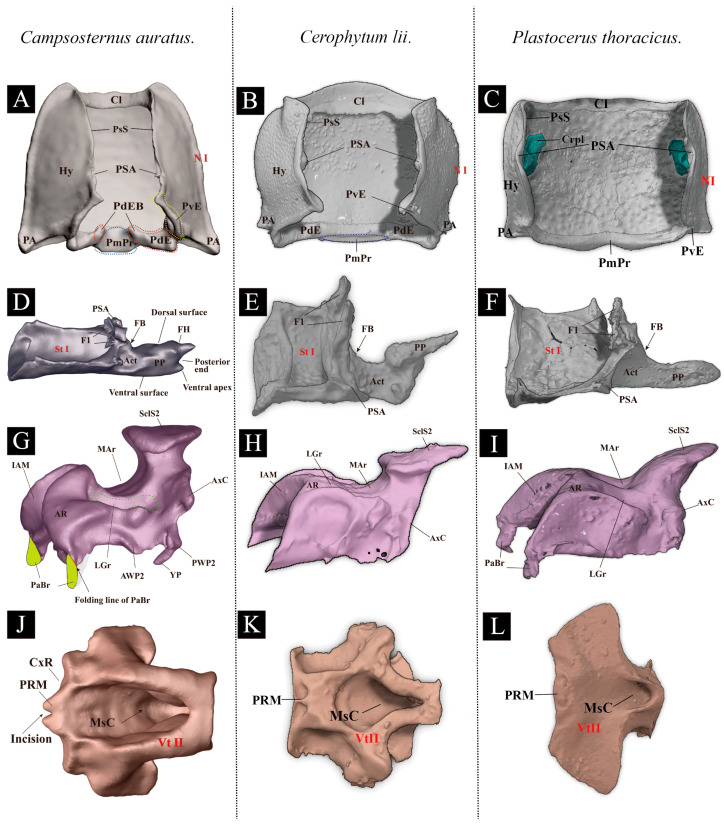
Comparison of the thoracic exoskeleton of *Campsosternus auratus, Cerophytum lii*, and *Plastocerus thoracicus*. (**A**–**C**): Ventral views of the pronotum of *Ca. auratus, Ce. lii,* and *P. thoracicus*. (**D**–**F**): Lateral views of the prosternum of *Ca. auratus, Ce. lii,* and *P. thoracicus*. (**G**–**I**): Lateral views of the mesonotum of *Ca. auratus, Ce. lii,* and *P. thoracicus*. (**J**–**L**): Ventral views of the mesoventrite of *Ca. auratus, Ce. lii*, and *P. thoracicus*; for technical information and abbreviations, see Section 2. Note: Partial annotations pertaining to *Cerophytum lii* have been corrected in this study.

**(1)**  **Pronotum (N I)** (Figure 7A–C)

*Ca. auratus*: The posterior angle of the pronotum (PA) is strongly developed. The posterior evaginations (PE) and posteromedial part (PmPr) of the pronotum are normal. The cryptopleuron (Crpl) is T-shaped.

*Ce. lii*: The PA, PE, and PmPr are all weakly developed. The Crpl is disc-shaped.

*P. thoracicus*: The PA, PE, and PmPr are weakly developed. The absence of the PdE results in a failure of the clicking mechanism. The Crpl is mushroom-shaped.

**(2)**  **Prosternum (Vt I)** (Figure 7D–F)

*Ca. auratus*: The prosternal process (PP) is strongly developed. The friction hold (FH) is well-developed. The prothoracic furca (F1) is fan-shaped, and the profurcal base (FB) is well-sclerotized.

*Ce. lii*: The PP is well-developed basally and weakly developed apically. The FH is absent. The F1 is fan-shaped, and the FB is well-developed.

*P. thoracicus*: The PP has a narrow base that expands abruptly into a teardrop-shaped apex, which is rounded and of uniform thickness. The FH is absent. The F1 is fan-shaped. The FB is obsolete.

**(3)**  **Mesonotum (N II)** (Figure 7G–I)

*Ca. auratus*: The median arched area of the mesonotum (MAr) is strongly developed. The lateral groove of the mesonotum (LGr) and the yoke plate (YP) are well-developed and closely associated with the metathoracic prescutum (Prs3). The anterolateral region of the mesonotum (AR) is the oblique and somewhat hemispherical area with a highly smooth surface; its dorsal surface articulates with the posterodorsal evagination (PdE). The mesoscutellar shield (SclS2) is significantly raised above. The mesoscutellar shield and lower part of the mesoscutellum fit with the mesal part of the elytra and form the elytral-mesoscutellar interlocking.

*Ce. lii*: The MAr is weakly developed. The LGr and YP are weakly developed. The AR is extremely weakly developed, oblique, and subhemispherical; the surface is highly smooth, and the dorsal margin conforms to PdE [13]. The SclS2 is raised above the surface of the mesonotum.

*P. thoracicus*: The mesonotum exhibits a sloping structure. MAr is extremely weakly developed, only slightly concave, and this configuration compromises the elastic storage of the clicking mechanism. The LGr is slightly concave and possesses two pits adjacent to the MAr. The YP is obsolete, which prevents a tight connection with the Prs3 and consequently leads to a weak or impaired clicking ability. The surface of AR is reduced, preventing the formation of a functional thoracic hinge joint with the PdE. The SclS2 and the MAr form a slope with an angle close to 45 degrees, whereas in other elaterids, its configuration is typically vertical.

**(4)**  **Mesoventrite (Vt II)** (Figure 7J–L)

*Ca. auratus*: The mesoventrite is deeply excavated. The mesoventral cavity (MsC) is cuneiform, and the prosternal rest of the mesoventrite (PRM) is V-shaped. The mesoventrite is deeply excavated in the ventral view. The mesoventrite has a well-developed MsC and PRM. The prosternal process (PP) is strongly developed and forms an interlocking structure with the PRM during the clicking process.

*Ce. lii*: The PRM is extremely weakly developed, only faintly traceable as an impression with a smooth surface [16]. The PP has poor contact with the PRM. The PRM is weakly developed.

*P. thoracicus*: The mesoventrite is broad overall and bears an extremely shallow MsC and PRM. The PP does not engage with PRM, which critically impairs the clicking functionality.

### 3.3. Comparative Morphology: Thoracic Musculature of Campsosternus auratus, Cerophytum lii, and Plastocerus thoracicus

*Ca. auratus*: The M2 muscle is normally developed, while the M4 muscle is highly developed and functions as the dominant muscle in the thorax and the primary site for energy storage. In contrast, the flight muscles (M60, M64) and walking muscles (M74, M75) are less developed compared to those in *Ce. lii*. The M12 is absent. (Volume ratio: M2/M60 = 0.16; M4/M60 = 1.66). (Table 1)

*Ce. lii*: The M2 and M4 muscles are weakly developed, whereas the muscles M60, M64, M74, and M75 are strongly developed. The M12 is well-developed. (Volume ratio: M2/M60 = 0.02; M4/M60 = 0.84). (Table 1)

*P. thoracicus*: The M2 and M4 muscles are weakly developed. The muscles M60, M64, M74, and M75 are exceptionally strongly developed. The M12 is well-developed. (Volume ratio: M2/M60 = 0.12; M4/M60 = 0.20). (Table 1)

**Table 1:** The thoracic musculature of *Selatosomus aeneus* [21]*, Campsosternus auratus, Cerophytum lii*, and *Plastocerus thoracicus*. Comparative analysis of muscular proportional representation within the body, with *Campsosternus auratus* as the reference standard. “○”= present; “-“= weakly developed; “+”= well-developed; “++”= strongly developed; “+++”extremely developed; “/”= absent; and “?”= unclear. (Figure 8 and Figure 9).

Larsén [21]	Friedrich and Beutel [32]	*Selatosomus* *aeneus*	*Campsosternus* *auratus*	*Cerophytum* *lii*	*Plastocerus* *thoracicus*
**M1**	**Idlm2**	○	+	+	+
**M2**	**Idlm1**	○	+	+ **(only M2b)**	+
**M3**	/	/	/	/	/
**M4**	**Idlm5**	○	+++	++	-
**M4x**	**Idlm5**	○	+	+	+
**M5**	**Ivlm3**	○	+	+	+
**M6**	**Ivlm1**	○	+	+	+
**M7**	**Idvm6**	○	+	?	+
**M8**	**Idvm8**	○	+	+	+
**M9**	**Idvm4?**	/	/	/	/
**M10**	**Idvm2,3**	○	+	+	?
**M11**	**Idvm10**	○	+	+	+
**M12**	**Itpm3**	○	/	+	+
**M13**	**Itpm6**	○	/	/	/
**M14**	**Idvm15**	/	/	/	/
**M15**	**Idvm16,17**	○	+	+	+
**M16**	**Ipcm4**	○	+	+	+
**M17**	**Ipcm6**	/	/	/	/
**M18**	**Iscm1**	/	/	/	/
**M19**	**Iscm2**	○	+	+	+
**M20**	**Ipcm8**	○	+	+	+
**M28**	**IIdlm1**	○	+	+	+
**M29**	**IIdlm2**	○	+	+	+
**M30**	**Ivlm7**	○	+	+	+
**M31**	**Ivlm9**	/	/	/	/
**M32x**	**IIdvm8?**	○	+	+	+
**M33**	**IItpm2**	○	+	+	+
**M34**	/	/	/	/	/
**M35**	**IItpm10**	○	/	/	/
**M36**	**IItpm9**	○	+	+	+
**M37**	**IIspm2**	○	+	+	+
**M38**	**Ispm6**	/	/	/	/
**M39**	**IIdvm2**	/	/	/	/
**M40**	**IIdvm4,5**	○	+	+	+
**M41**	**IIpcm4**	○	+	+	+
**M42**	**IIpcm2**	/	/	/	/
**M43**	**IIdvm6**	/	/	/	/
**M44**	**IIscm3**	○	/	/	/
**M45**	**IIscm4**	/	+	+	+
**M46**	**IIscm2**	○	+	?	+
**M47**	**IIdvm3**	/	/	/	/
**M48**	**IIpcm6**	○	+	+	+
**M49**	/	/	/	/	/
**M50**	**IIpcm5?**	/	/	/	/
**M51**	/	/	/	/	/
**M52**	**IIscm6**	○	/	/	/
**M60**	**IIIdlm1**	○	+	++	+++
**M61**	**IIIdlm2**	○	+	+	+
**M62**	**IIvlm3**	○	/	+	/
**M63**	**IIvlm5**	/	/	/	/
**M64**	**IIIdvm1**	○	+	++	+++
**M65**	/	○	/	/	/
**M66**	**IIIdvm8**	○	+	+	+
**M67**	**IIItpm2**	○	+	+	+
**M68**	**IIItpm11**	/	/	/	/
**M69**	**IIItpm3**	○	+	+	+
**M70**	**IIItpm10**	○	/	/	/
**M71**	**IIItpm7,9**	○	+	+	+
**M72**	/	○	/	/	/
**M73**	**IIIspm1**	○	+	+	+
**M74**	**IIIdvm2**	○	+	++	+++
**M75**	**IIIdvm4**	○	+	++	+++
**M76**	**IIIdvm5**	○	+	+	+
**M77**	**IIIpcm4**	/	/	/	/
**M78**	**IIIpcm3**	○	+	+	+
**M79**	**IIIdvm6**	○	+	+	+
**M80**	/	/	/	/	/
**M81**	**IIIscm1**	○	+	+	+
**M82**	**IIIscm4**	○	+	+	+
**M83a**	**IIIscm2**	○	+	+	+
**M83b**	**IIIscm3**	○	+	+	+
**M84**	**IIIdvm3**	/	/	/	/
**M85**	**IIIscm6**	○	+	+	+

Note: Qiu et al. [16] did not mention M12 in *Cerophytum lii*, but its presence is confirmed in this study.

**Figure 8:**
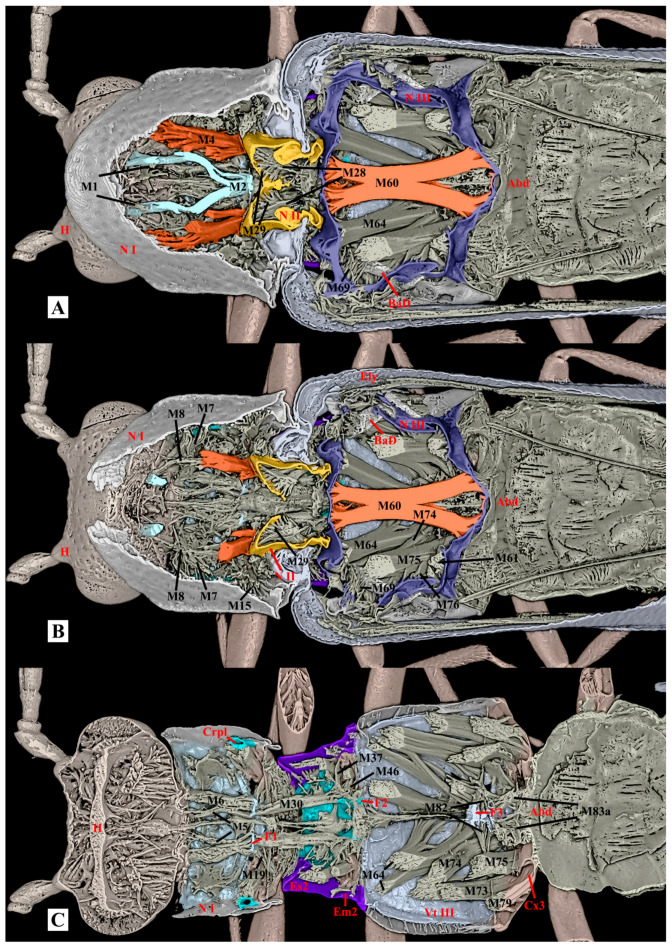
Three-dimensional reconstructions of the thoracic exoskeleton and musculature of *Plastocerus thoracicus*, dorsal view. The head of the model is facing left, and the exoskeleton of the model is cut in the frontal (coronal) plane at different layers (**A**–**C**) to show internal musculature; for technical information and abbreviations, see Section 2.

**Figure 9:**
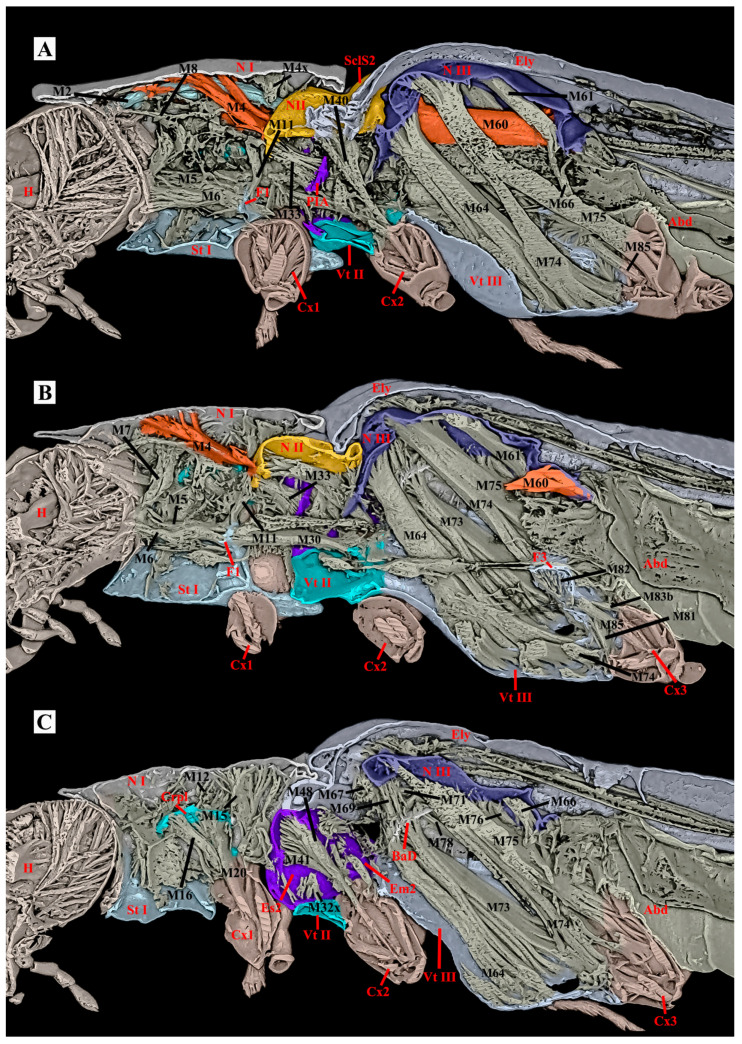
Three-dimensional reconstructions of the thoracic exoskeleton and musculature of *Plastocerus thoracicus*, lateral view. The head of the model is facing left, and the exoskeleton of the model is cut in the sagittal (**A**) and parasagittal (**B**,**C**) planes at different layers to show internal musculature; for technical information and abbreviations, see Section 2.

## 4. Discussion

In this study, we investigated the sclerites and muscles of the thorax in *Plastocerus thoracicus* and revealed several primary feature changes that are related to its soft-bodiedness and the absence of a clicking mechanism:

(1) **Weakened M4 Muscle.** In other click beetles, the M4 muscle drives ventral bending of the pronotum, which establishes and releases elastic potential energy for clicking. The extreme weakness of M4 in *Plastocerus thoracicus* renders it functionally inadequate, and the comparative volume of the M4 in this species is somewhat close to other beetles without clicking apparatus, such as the bark beetle *Xylosandrus amputatus* (Blandford) (Curculionidae: Scolytinae) [33]. In contrast, the M4 is fully developed in *Campsosternus auratus*, ensuring a powerful clicking mechanism. In *Cerophytum lii*, the M4 exhibits partial reduction but retains basic clicking functionality.
(2) **Loss of Thoracic Hinge System**. In a normal clicking system, the posterodorsal evagination (PdE) must form a functional hinge with the anterolateral region of the mesonotum (AR). The development of the PdE and AR varies among species: they are underdeveloped in *Plastocerus* thoracicus, preventing hinge formation; well-developed in *Campsosternus auratus*, ensuring stability; and partially functional in *Cerophytum lii*.
(3) **Loss of Biological Spring and Elytral-Scutellum Interlocking Mechanism**. In common clicking beetles, the N II forms a delicate saddle-shaped biological spring, and the elevated mesoscutellar shield (SclS2) and the median-arched area (MAr) form interlocking structures with elytra [4]. Both the spring and interlocking structures are absent in *Plastocerus thoracicus;* they are fully developed in *Campsosternus auratus* (optimal function), and they are slightly weakened in *Cerophytum lii*.
(4) **Loss of Loading and Triggering Structures**. A functional clicking requires Loading and Triggering of the system. The prosternal process (PP) and the mesoventral cavity (MsC) engagement requires the robust morphology of PP, the friction hold (FH), and the deep MsC. However, *Plastocerus thoracicus* exhibits slender PP, absent FH, and shallow MsC, preventing engagement of a proper Loading process; *Cerophytum lii* has a weakened clicking function due to a narrow PP apex and the absence of FH.
(5) **Enhancement of Flight and Walking Muscles.** The flight muscles (M60, M64) and walking muscles (M74, M75) exhibit species-specific developmental gradients: they are extremely strong in *Plastocerus thoracicus*, strongly developed in *Cerophytum lii*, and moderately developed in *Campsosternus auratus*.

Furthermore, the M12 muscle are present in both *Cerophytum lii* and *Plastocerus thoracicus* but absent in *Campsosternus auratus*. According to Larsén’s study [21], the M12 muscle is present in *Selatosomus aeneus* and is commonly found in Carabidae, some Dytiscidae, and all polyphagan groups characterized by an apical cryptopleuron that remains unfused with the notum. We hypothesize that *Plastocerus thoracicus* and *Cerophytum lii* possess relatively strong walking and locomotor capabilities, an inference consistent with the results reported in Qiu et al. [16]. Their work pointed out that longer legs indicate enhanced walking capability [16]. This morphological pattern indicates that *P. thoracicus* prioritized energy investment in flight and fast walking locomotion rather than the clicking mechanism, whereas *Ca. auratus* adopted a distinct evolutionary strategy focused on refining its clicking musculature. Beyond the functional morphological significance, this study provides crucial morphological data that may help clarify the taxonomic position of *Plastocerus* in future research. Furthermore, a broader comparative investigation of potentially related elaterid groups is warranted, as it may yield additional morphological evidence to help establish the genus’s phylogenetic position.

## 5. Conclusions

In this study, the thoracic morphology of *Plastocerus thoracicus* is studied and compared with that of *Campsosternus auratus* (Elateridae) and *Cerophytum lii* (Cerophytidae), with emphasis on the structures associated with the clicking mechanism. We reveal that the reduction in numerous clicking-related sclerites and muscles in *Plastocerus thoracicus* collectively accounts for the loss of its clicking ability. This functional loss reflects a significant shift in ecological strategy: although reduced jumping capacity decreases the probability of escaping predation, the conserved energy appears to have been reallocated to enhance other adaptive traits, such as flight proficiency, walking efficiency, or potentially the development of defensive structures (e.g., possible chemical defenses). These compensatory adaptations may ultimately improve its survival advantage in specific habitats.

This study provides critical morphofunctional insights into the loss of clicking mechanisms in elaterid beetles and offers a new perspective on the relationship between insect morphological evolution and niche differentiation. Several other elaterid groups, such as Agrypninae—Drilini—and Omalisinae also exhibit loss of clicking ability and even more extreme body softening than *Plastocerus*. Future comparative studies on these taxa would further illuminate the evolutionary mechanisms behind this phenomenon.

## Data Availability

The original contributions presented in this study are included in the article. Further inquiries can be directed to the corresponding authors.

## Copyright Permission

© 2026 All Authors and His Majesty the King in right of Canada as Represented by the Minister of Agriculture and Agri-Food Canada.
